# Controlling neuronal assemblies: a fundamental function of respiration-related brain oscillations in neuronal networks

**DOI:** 10.1007/s00424-022-02708-5

**Published:** 2022-05-31

**Authors:** Shani Folschweiller, Jonas-Frederic Sauer

**Affiliations:** 1 Institute for Physiology I, Medical Faculty, Albert-Ludwigs-University Freiburg, Hermann-Herder-Strasse 7, 79104 Freiburg, Germany; 2 Faculty of Biology, Albert-Ludwigs-University Freiburg, Schaenzlestrasse 1, 79104 Freiburg, Germany

**Keywords:** Breathing, Respiration, Neuronal assembly, Cortex

## Abstract

Respiration exerts profound influence on cognition, which is presumed to rely on the generation of local respiration-coherent brain oscillations and the entrainment of cortical neurons. Here, we propose an addition to that view by emphasizing the role of respiration in pacing cortical assemblies (i.e., groups of synchronized, coactive neurons). We review recent findings of how respiration directly entrains identified assembly patterns and discuss how respiration-dependent pacing of assembly activations might be beneficial for cognitive functions.

## Introduction

Due to the biological necessity of repeatedly alternating expiration and inspiration to ensure the constant supply of oxygen and the evacuation of metabolically produced CO_2_, respiration brings with it the properties of a continuously repeating timing signal. Originating in mechanosensitive olfactory sensory neurons that are driven by airflow in the nasal cavity and transmit their activity to the olfactory bulb [2, 29], a large body of evidence supports the notion that respiration-related oscillations (RROs) occur all over the neocortex [5, 6, 27, 30, 36, 39, 41, 43, 52, 67, 68, 73], hippocampus [39, 46, 55], and other subcortical structures [39]. The respiration-synchronous rhythm is ubiquitously present in the forebrain during offline states [27, 39, 68] and is thus an excellent candidate to induce transient periods of synchrony between distant brain regions. Emerging evidence supports the idea that respiration plays a key role in this coordination across multiple brain regions: single-unit responses to hippocampal sharp wave ripples in the mouse medial prefrontal cortex (mPFC) depend robustly on the phase of the respiration at which these hippocampal transient increases in excitability occur [39]. Furthermore, respiration-coherent 4-Hz rhythms synchronize the mPFC and the amygdala during fear-related freezing in rodents [5, 38, 39, 52]. Based on the idea that the timing and synchronization provided by the respiratory cycle are behaviorally relevant, human memory encoding, retrieval, and perception [3, 40, 54, 71] have been shown to be modulated by the phase of respiration. These results suggest a profound impact of respiration on cognition [33].

**Fig. 1:**
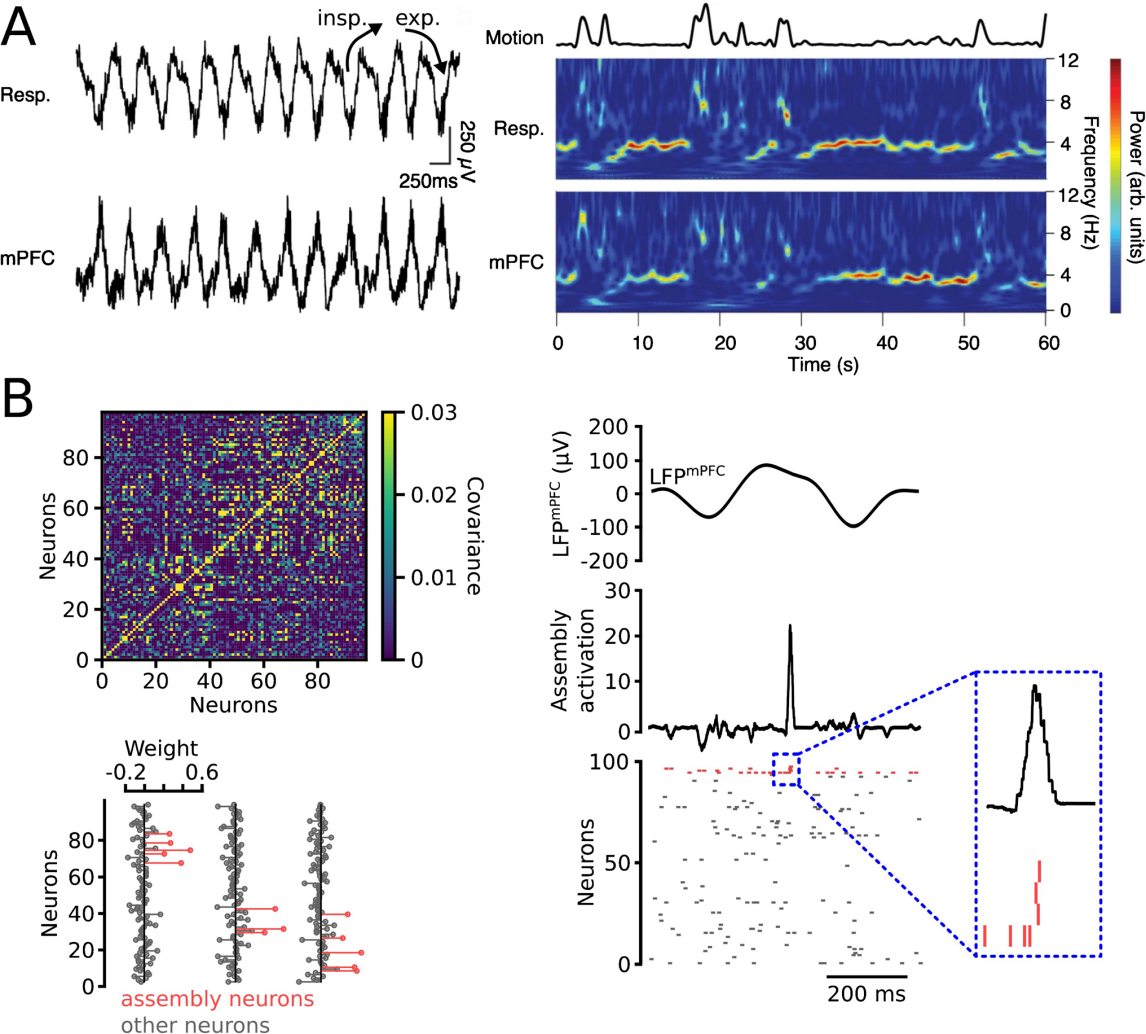
Detecting RROs and assemblies in the neocortex. **A** RROs are observed as local field potential oscillations that occur synchronized with nasal respiration. Nasal breathing is often assessed by electroolfactogram [24, 39] (i.e., electrical recording from the nasal epithelium as shown in the example on the left along with local field potential measurement from the mPFC). Alternatively, body-plethysmography [67], thermocouples [73] or pressure sensors [46] implanted in the nasal cavity, or piezoelectric sensors recording chest movement [70] (in anesthetized preparations) are commonly used. Right: Respiration-coherent 2–4 Hz RROs emerge in the mPFC during immobility. The example shows power spectral density of the electrical activity in the nasal epithelium (top) and mPFC (bottom). Adapted from [39] under CC BY 4.0 (https://creativecommons.org/licenses/by/4.0/). **B** Assemblies can be detected by calculating the corresponding weight vectors for all single units. Bottom left: example of weight vectors of prefrontal single units for three simultaneously recorded assemblies. The neurons are arranged by order of appearance along a linear probe inserted along the dorso-ventral axis of the mPFC. These vectors can be extracted from covariance matrices (top left) using independent component analysis. See [48] for review of assembly extraction methods. Assembly time courses (right middle) are estimated from the weight vectors and the underlying spike train (right, bottom) and can be correlated to ongoing RROs (or other field potential oscillations, shown on top). Neurons contributing to the example pattern are shown in red. Adapted from [24] under CC BY 4.0

While the mechanisms of cognition are not resolved and subject of ongoing research, a key concept dating back more than 70 years is that of the *cell assembly,* which postulates that associations of coactive neurons rather than individual cells form the unitary operational entity of cortical functions [32]. RROs indeed not only modulate the spiking of rodent cortical neurons [5, 6, 37, 39, 43, 55] but also, as postulated by Tort et al. in 2018 [68], have recently been directly demonstrated to pace the emergence of electrophysiologically identified cortical assemblies [24] (Fig. 1), suggesting that RROs might affect cognition by defining when and where assemblies activate. In this review, we will lay out recent findings in support of the assembly-centric view on RROs.

## Neuronal assembly theory

The basic concept of the assembly is attributed to Donald Hebb [32] and has been discussed in detail elsewhere (see [9, 31, 57, 61]). In brief, Hebb’s assembly theory arose from observations such as the fact the brain is able to hold memories permanently and can generalize perceptions, that cognitive functions are often intact even after substantial loss of neurons, and that behavior cannot be explained by the immediately preceding sensory stimulation alone. Thus, he argued, rather than relying on single neurons, *assemblies* of neurons would develop after the repeated and conjoined activation of groups of neurons within a brain structure, which would then be stabilized by plastic changes at their mutual synapses. The notion of the assembly emphasizes the strengthening of synaptic connections between coactive neurons (or the emergence of new connections, see [17] for review) such that partial activation of a subset of the contributing neurons could “ignite” the entire assembly (this notion is similar to the concept of pattern completion by recurrent connections among pyramidal neurons in the hippocampal CA3 region [51]). Moreover, an assembly could then activate downstream assemblies, essentially chaining active groups of neurons in what Hebb called the *phase sequence*.

Besides a plethora of theoretical work (e.g., [1, 7, 26, 64]), evidence for the presence of assemblies has indeed been found experimentally in non-human research models: coactivity among neocortical pyramidal cells induces “Hebbian” strengthening of their synaptic connections in vitro [50], and groups of neocortical neurons that repeatedly coactivate have been observed in vivo [21, 23, 24, 47, 48, 56, 60, 69]. While these transient synchronizations of assembly neurons are thought to be due to reciprocal synaptic connectivity, one should keep in mind that synchronous presynaptic activity might contribute as well [8]. Nonetheless, coactive neurons within the mPFC are close together in space [24] (Fig. 1B), which is consistent with the decay in the probability of synaptic connections between pyramidal neurons in the cortex with distance [11]. Moreover, in a recent imaging study, Carrillo-Reid et al. used holographic stimulation to induce coactivity in a subset of visual cortex neurons [14]. After this “imprinting” step, the stimulated neurons retained their tendency to coactivate spontaneously. While it can be argued about whether or not optogenetically induced “photoensembles” reflect bona fide assemblies, they show similar properties to naturally occurring groups of coactive cells recorded under the same conditions [12, 14]. Stimulating single neurons that are part of an imprinted “photoensemble” moreover resulted in the activation of the ensemble, confirming that artificially generated ensembles possess pattern completion capability [14]. These findings thus validated key predictions of Hebb’s original proposal and put experimental weight behind the theoretical concept of the cell assembly.

Buzsaki (2010) argued that assemblies should be defined by looking at downstream “reader” systems [9]. In this framework, the conjoint activity of single neurons is necessary to successfully transmit information to the adequate downstream reader. Consider a neocortical neuron that receives synaptic input from upstream pyramidal neurons as such a reader system. Due to the low efficacy of synaptic connections among pyramidal cells (for a recent measurement of the amplitude of excitatory postsynaptic potentials at layer 2/3 pyramidal-pyramidal connections in mouse (~ 0.11 mV) and human cortex (~ 0.2–0.4 mV), see [11]), the reader cell will only respond with a spike of its own if a sufficiently large number of upstream neurons fire synchronously. How synchronous do upstream action potentials need to be? The membrane time constant of mammalian cortical pyramidal cells (~ 10–30 ms [42]) defines a prevailing time window during which the summation of excitatory postsynaptic potentials can trigger a spike [9, 66], although the time constant can vary [42]. Anatomical constraints such as the location of the synapses of an upstream assembly on the dendritic tree of the target cell as well as dendritic integration properties might further limit the capability of a set of presynaptic neurons to induce spiking in the reader [44, 62, 66]. However, as addressed by a recent study in rodents [56], the majority of cell assemblies activates within a 10-ms time window, and increasing the window of what is considered synchronous activity up to 150 ms led only to minor increases in the number of detected assemblies [19, 56]. Since assembly neurons are required to activate synchronously to jointly drive postsynaptic (reader) neurons, they can be considered a quasiuniform presynaptic entity. The assembly thus solves the problem of weak individual synapses and has therefore been hypothesized to be a key component of cortical function.

## Respiration as an organizer of assembly activation

Emerging evidence from rodent studies supports the notion that RROs might play an important role in defining when neuronal assemblies become active: a recent preprint showed that olfactory neuronal assemblies in the mouse piriform cortex, the primary olfactory area receiving direct input from the olfactory bulbs, are paced by respiration [28]. The first account of functionally defined prefrontal assemblies being modulated by putative RROs came from a study by Dejean et al. [19]. They detected assemblies as coactive neurons in the mouse mPFC (within 150-ms time bins) using principal component analysis and found that assemblies activated specifically on the ascending phase of putative RROs during the expression of conditioned fear (i.e., freezing in response to a tone that had been previously paired with a foot shock [19]). We cautiously speak of putative RROs here since the authors did not directly measure respiration (or a suitable proxy such as the local field potential in the nasal epithelium [39]) but rather 4-Hz oscillations that emerge during freezing in mPFC and amygdala of mice [38]. However, several studies using distinct methods of respiration recording have since demonstrated that these freezing-related 4-Hz oscillations are highly coherent with nasal respiration [5, 39, 52]. Four-Hertz oscillations in the rodent mPFC are moreover greatly attenuated when transmission from the olfactory bulb to the cortex is blocked using optogenetics [5] or pharmacologically [52], so we can conclude with high confidence that the reported association between assembly activity and ongoing 4-Hz activity reflects the entrainment of assemblies by RROs. These results thus emphasize an enrichment of assembly activations during the ascending RRO phase in a specific behavioral state associated with the expression of learned fear.

**Fig. 2:**
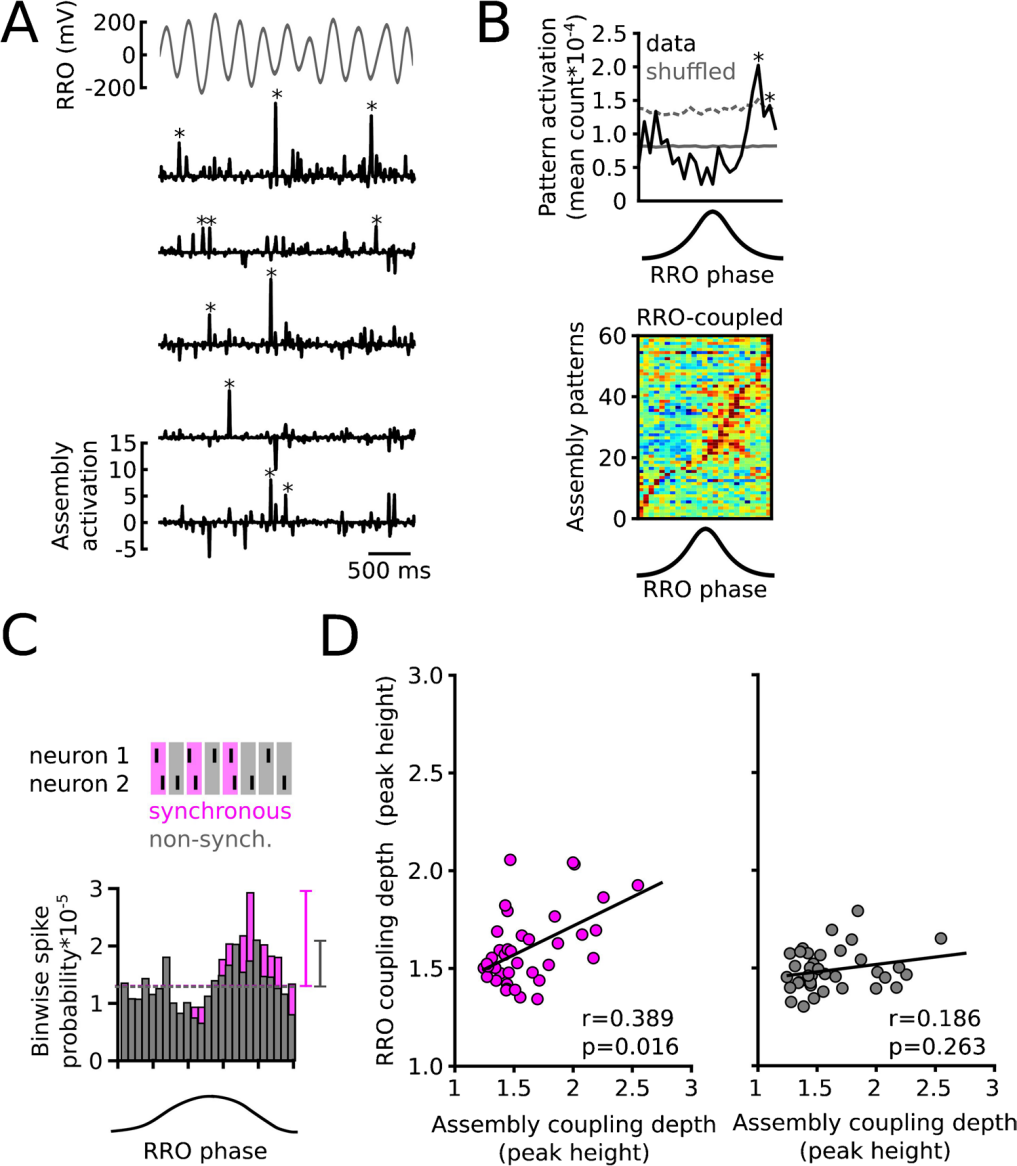
Respiration-paced assemblies in the mPFC. **A** Example of ongoing RRO (top) and activation time courses of five assembly patterns during spontaneous immobility. Asterisks mark significant (> 5SD) assembly activations. **B** During spontaneous immobility, assemblies are biased toward activating during the descending RRO phase. Top: example pattern activation as a function of RRO phase. Dashed line: significance threshold. Bottom: summary of significantly entrained assemblies. **C** Synchronous spikes between assembly member neurons occur more often during the descending RRO phase than non-synchronous spikes. **D** Coupling depth of synchronous spikes of member neurons (left) correlates more strongly with the coupling depth of assemblies than that of non-synchronous spikes (right), suggesting that neuronal coactivity is paced by RROs. Adapted from [24] under CC BY 4.0 (https://creativecommons.org/licenses/by/4.0/)

Calcium imaging data from visual cortex found that neuronal assemblies occur not only in relation to specific stimuli but also in the absence of stimulation (i.e., spontaneously [13]). Based on that result and the aforementioned specific activation of assemblies during the ascending RRO phase during fear expression, we recently asked whether RROs might be utilized to organize assembly activations in the absence of specific stimuli [24]. We recorded spontaneously occurring assemblies in the mPFC of mice using a PCA/ICA framework [48] to track not only the time course of assembly activations but to also identify the contributing neurons. Assemblies were indeed detected during spontaneous immobility, but unlike in the case of fear expression, these coactivity patterns emerged at higher frequency during the descending rather than the ascending phase of RRO (Fig. 2). Interestingly, while the RRO-coupling depth of individual neurons positively correlated with the coupling depth of the assemblies they contributed to, the coupling of assemblies to RROs was not simply a function of the RRO pacing of contributing neurons: when we measured the RRO coupling only using spikes that occurred synchronously (i.e., within 10 ms) with the spikes of other members of the same assembly, we found stronger RRO coupling of synchronous spikes and a correlation to the coupling strength of the assembly while the same was not true for the correlation between assembly and neuron coupling strength using non-synchronous spikes (Fig. 2). Stronger RRO coupling of synchronous spikes may arise from a lower spike threshold observed under conditions of rapidly depolarizing membrane potential [4, 34], which might come about during descending RRO due to synchronous respiration-paced long range as well as recurrent local inputs from highly active mPFC neurons [24]. While the mechanisms remain unclear, these findings suggest that on top of biasing the time of firing of individual neurons, the neuronal coactivity itself is paced by ongoing RROs. These results bear similarity to a recently described “coactivity code” in the hippocampal CA1, in which learned behavioral contingencies can be decoded from short timescale correlations among pyramidal cells [21].

In addition to the direct observations of assemblies during RROs, several indirect pieces of evidence further support the role of respiration in organizing assembly activations. First, sharp wave ripples, which occur during offline states in the hippocampus as well as in the mPFC and represent the synchronous activity of pyramidal cells, are entrained by respiration [39, 45]. During ripples, behaviorally relevant spike sequences that occur on the seconds time scale are replayed in a compressed manner [20, 25]. It has been argued that Hebbian plasticity during replay might strengthen connections among neurons [53], which could facilitate the formation of new assemblies. In this model, RROs might support the emergence of new assemblies by offering the time windows for Hebbian “binding” of the assembly [53]. However, due to the large difference in time scale of the sequence during behavior and replay, it remains unclear to what extent replay events reflect classical cell assemblies. Second, gamma oscillations offer a time window of activation of 10–40 ms, which fits the prevailing time of assembly activation that we discussed earlier. Phase-amplitude coupling between gamma and RRO seems to be a ubiquitous feature in both cortical and subcortical areas [39, 73] and across species [15, 35, 41], which might be an indication of a more widespread entrainment of neuronal assemblies by RROs. Using a strict analytical procedure, one study showed explicitly that the activity of assembly neurons occurs coupled to ongoing gamma oscillations in the rat mPFC [56]. In addition, a recent preprint demonstrated a link between the power of respiration-paced gamma oscillations and the activity of assembly neurons [28]. Interestingly, assembly neurons in the piriform cortex of mice showed reduced gamma phase coupling compared to non-assembly neurons, suggesting a role of gamma in suppressing the activity of competing assemblies [28]. Further work is required to understand the extent and the underpinnings of the link between RROs, gamma oscillations, and assemblies: for example, as suggested before [33], respiration measurements should be routinely included in experiments utilizing large-scale single-unit and local field potential recordings. That way, respiration-entrained gamma oscillations could be directly measured alongside the activity of assemblies during distinct tasks, which might bring important insights into the ongoing computation ruling assembly activation.

## Potential functional implications of respiration-paced assembly activations

Dejean et al. provided a direct assessment of the functional importance of 4-Hz rhythmic assemblies for fear expression: they optogenetically drove parvalbumin-positive interneurons during the ascending phase of 4-Hz oscillations recorded in the mPFC [19]. This manipulation reduced the firing rate and coactivity of assembly neurons specifically during ascending RRO and significantly attenuated freezing, while applying the same manipulation during the descending phase increased freezing. Based on these results, the authors proposed that mPFC assemblies active during the ascending phase might efficiently drive downstream receivers mediating the freezing response, potentially via the amygdala. The amygdala is of central importance for fear responses and has been shown to synchronize with the mPFC at 4 Hz during freezing [38]. The authors provide a compelling model in which amygdala neurons might be able to read out a freezing command from prefrontal assembly activity during the ascending RRO phase. Importantly, this drive to the amygdala might emerge from background activity due to the enhanced efficacy of synaptic drive of synchronously active assembly neurons.

**Fig. 3:**
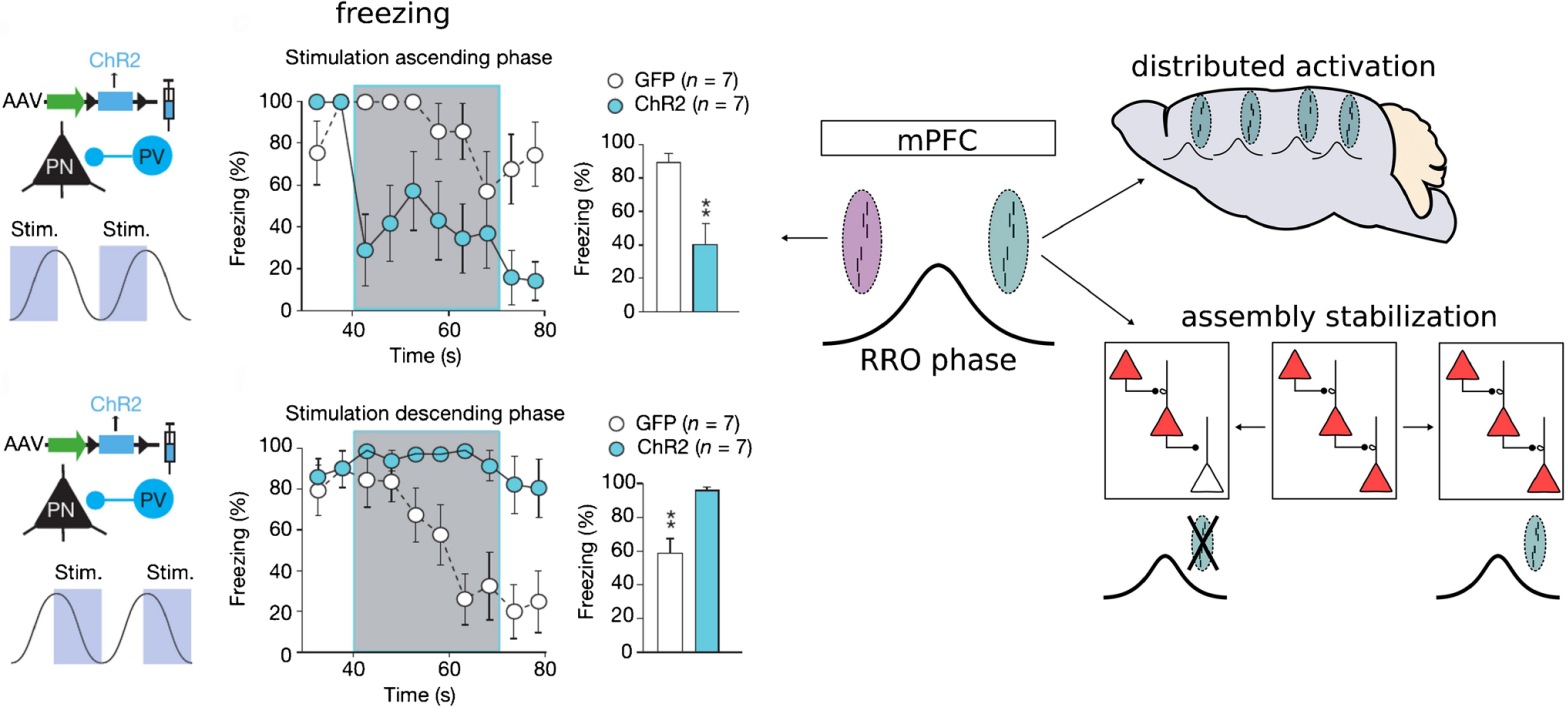
Model of the proposed functional implications of respiration phase-entrained assembly activations. Left: during recall of conditioned fear, prefrontal assemblies activate during the ascending phase of RROs and control behavioral output (i.e., freezing) [19], potentially through synchronization with the amygdala [38]. Silencing assemblies by the activation of channel rhodopsin 2 (ChR2)-expressing parvalbumin-positive interneurons reduces (top) and enhances freezing (bottom) when the manipulation is targeted to the ascending and descending phase of 4-Hz oscillations, respectively, suggesting that assemblies nested in the ascending 4-Hz phase drive the expression of freezing [19]. Adapted by permission from Springer Nature, Nature, Dejean et al. 2016, copyright (2016). Right: during spontaneous immobility, assembly activations occur more frequently during the descending phase [24], which might aid the “ignition” of distributed assemblies due to the widespread phase synchrony of RROs (top) or support the spontaneous reactivation of assemblies to counteract the time-dependent decay of the assemblies due to spine turnover (bottom)

In the absence of fear expression, RRO-coupled assemblies appear enriched in the descending prefrontal RRO phase. RROs might thus provide distinct time windows for sender-reader interactions: assemblies nested in the ascending phase could be important for instructing freezing (by driving amygdala neurons) [19], while assemblies nested in the descending phase could reflect a “default state” which might aid the widespread activation of distributed assemblies (which might not specifically involve the amygdala, Fig. 3). Interestingly, the overall phase of RROs seems conserved across the olfactory bulb, the amygdala, the nucleus accumbens, and even across layers of the mPFC [39], with only approximately 20° of phase shift between RROs in the mPFC and in the amygdala [38], indicating that the descending phase measured in one of these structures corresponds broadly to the same phase in the others. Nonetheless, the notion of distinct RRO phases being utilized as different communication channels is speculative and warrants further investigation. It is, however, in principle plausible: in the hippocampal CA1, gamma oscillations reflecting distinct input streams (low-range gamma from CA3 and mid-range gamma from the entorhinal cortex [18, 63]) recruit different ensembles of neurons [49]. Importantly, these distinct gamma activities are nested in the ongoing theta rhythm in a phase-specific manner, namely with slow gamma occurring during the descending phase of theta and mid-range gamma around the peak [49, 63]. Without claiming that the mechanisms of the phase-specific recruitment of assemblies to RROs resemble those of gamma-modulated CA1 inputs, it will be interesting to further explore the idea of behavior-dependent and RRO phase-defined communication streams among cortical areas.

Driving assemblies during the descending RRO phase could be beneficial for an additional reason. Recent imaging data showed substantial stability of some neuronal ensembles over several weeks [59], and theoretical work proposed the spontaneous reactivation of assemblies as a potential stabilization mechanism [22]. “Offline reactivation” of assemblies could effectively counter the slow loss of assembly members over time. This loss is expected since cortical synapses undergo constant remodeling such that new connections are formed and established connections are lost (for review see [16]). This implies that assemblies, once formed via a Hebbian mechanism, should destabilize over time due to spine turnover. In the Hebbian framework, repeated “ignitions” of the assembly are expected to strengthen the existing synapses among the members. An efficient way of offline reactivation is during hippocampal sharp wave ripples, which has been demonstrated for behaviorally defined assemblies in the hippocampus [69] and prefrontal cortex in rodents [60]. The favored activation of assemblies during the descending RRO phase under “default” conditions might provide an additional mechanism to support that stabilization of assemblies over relevant time scales on the cellular level by allowing “ignitions” of the assembly. In that view, the rhythmic depolarization provided by RROs would be simply a way to maximize the synchronous activation of neurons during the descending phase.

## Outlook and future directions

The phase of respiration makes a difference for cognitive function, including perception and memory encoding/retrieval in humans, but how the respiratory “timing signal” achieves that mechanistically is not understood. Rodent studies allow invasive recordings and have thus been utilized to reveal a detailed picture of the entrainment of fast brain rhythms and assemblies by RROs. However, there still is a large gap between (cognitive) human and (mechanistic) rodent data that will need bridging in future. Respiration frequency differs substantially across species: At rest, it averages at ~ 3 Hz in mice [73], ~ 0.2 Hz in cats [15, 72], and ~ 0.3 Hz in humans [35, 41]. These findings raise the question whether assemblies in the human neocortex are entrained by respiration, and whether similar phase-specific pacing mechanisms as observed in mice occur. Faster oscillatory frequencies (e.g., gamma oscillations) and action potential kinetics are largely preserved across species [10] but direct observations of assembly dynamics in the human cortex have to our knowledge not been reported. The advent of intrasurgical large-scale recording techniques [58], however, should enable investigations of assemblies and their relation to ongoing respiration in the human cortex in the future. As a complementary approach, future rodent experiments could combine multi-site large-scale recordings during cognitive tasks [65] to better understand the role of respiration-paced assembly activations on a more global scale. Particularly important aspects of the latter approach would be to better define the extent of assemblies and their task-related formation: assemblies have been reported to span across structures [56], but carefully conducted analyses are required to distinguish between assemblies with spatially distributed members and phase sequences. In addition, less than 2% of prefrontal neurons are part of assemblies at rest [24], while during cognitive tasks that engage the mPFC network, approximately one-third of the population assumes assembly membership [56]. Considering the clear role of respiration in cognitive processes, dissecting out the functional properties of distributed assemblies and phase sequences as a function of respiratory phase is a promising strategy to narrow down how rhythmic breathing might shape cognition.

## Data Availability

Data sharing not applicable to this article as no datasets were generated or analyzed during the current study.

## References

[1] Abeles M, Bergman H, Gat I, Meilijson I, Seidemann E, Tishby N, Vaadia E (1995). Cortical activity flips among quasi-stationary states. PNAS.

[2] Adrian ED (1942). Olfactory reactions in the brain of the hedgehog. J Physiol.

[3] Arshamian A, Iravani B, Majid A, Lundström JN (2018). Respiration modulates olfactory memory consolidation in humans. J Neurosci.

[4] Azouz R, Gray CM (2000). Dynamic spike threshold reveals a mechanism for synaptic coincidence detection in cortical neurons in vivo. Proc Natl Acad Sci.

[5] Bagur S, Lefort JM, Lacroix MM, de Lavilléon G, Herry C, Chouvaeff M, Billand C, Geoffroy H, Benchenane K (2021). Breathing-driven prefrontal oscillations regulate maintenance of conditioned-fear evoked freezing independently of initiation. Nat Commun.

[6] Biskamp J, Bartos M, Sauer J-F (2017). Organization of prefrontal network activity by respiration-related oscillations. Sci Rep.

[7] Braitenberg V (1978) Cell assemblies in the cerebral cortex. In: Heim R, Palm G (eds) Proceedings Symposium on theoretical approaches to complex systems 1977. Lecture notes in biomathematics

[8] Brama H, Guberman S, Abeles M, Stern E, Kanter I (2015). Synchronization among neuronal pools without common inputs: in vivo study. Brain Struct Funct.

[9] Buzsáki G (2010). Neural syntax: cell assemblies, synapsembles, and readers. Neuron.

[10] Buzsáki G, Logothetis N, Singer W (2013). Scaling brain size, keeping timing: evolutionary preservation of brain rhythms. Neuron.

[11] Campagnola L, Seeman SC, Chartrand T, Kim L, Hoggarth A, Gamlin C, Ito S, Trinh J, Davoudian P, Radaelli C, Kim M-H, Hage T, Braun T, Alfiler L, Andrade J, Bohn P, Dalley R, Henry A, Kebede S, Alice M, Sandman D, Williams G, Larsen R, Teeter C, Daigle TL, Berry K, Dotson N, Enstrom R, Gorham M, Hupp M, Dingman Lee S, Ngo K, Nicovich PR, Potekhina L, Ransford S, Gary A, Goldy J, McMillen D, Pham T, Tieu M, Siverts L, Walker M, Farrell C, Schroedter M, Slaughterbeck C, Cobb C, Ellenbogen R, Gwinn RP, Keene CD, Ko AL, Ojemann JG, Silbergeld DL, Carey D, Casper T, Crichton K, Clark M, Dee N, Ellingwood L, Gloe J, Kroll M, Sulc J, Tung H, Wadhwani K, Brouner K, Egdorf T, Maxwell M, McGraw M, Pom CA, Ruiz A, Bomben J, Feng D, Hejazinia N, Shi S, Szafer A, Wakeman W, Phillips J, Bernard A, Esposito L, D’Orazi FD, Sunkin S, Smith K, Tasic B, Arkhipov A, Sorensen S, Lein E, Koch C, Murphy G, Zeng H, Jarsky T (2022). Local connectivity and synaptic dynamics in mouse and human neocortex. Science.

[12] Carrillo-Reid L, Han S, Yang W, Akrouh A, Yuste R (2019). Controlling visually guided behavior by holographic recalling of cortical ensembles. Cell.

[13] Carrillo-Reid L, Miller JK, Hamm JP, Jackson J, Yuste R (2015). Endogenous sequential cortical activity evoked by visual stimuli. J Neurosci.

[14] Carrillo-Reid L, Yang W, Bando Y, Peterka DS, Yuste R (2016). Imprinting and recalling cortical ensembles. Science.

[15] Cavelli M, Castro-Zaballa S, Gonzalez J, Rojas-Líbano D, Rubido N, Velásquez N, Torterolo P (2020). Nasal respiration entrains neocortical long-range gamma coherence during wakefulness. Eur J Neurosci.

[16] Chambers AR, Rumpel S (2017). A stable brain from unstable components: emerging concepts and implications for neural computation. Neuroscience.

[17] Chklovskii DB, Mel BW, Svoboda K (2004). Cortical rewiring and information storage. Nature.

[18] Colgin LL, Denninger T, Fyhn M, Hafting T, Bonnevie T, Jensen O, Moser M-B, Moser EI (2009). Frequency of gamma oscillations routes flow of information in the hippocampus. Nature.

[19] Dejean C, Courtin J, Karalis N, Chaudun F, Wurtz H, Bienvenu TCM, Herry C (2016). Prefrontal neuronal assemblies temporally control fear behaviour. Nature.

[20] Diba K, Buzsáki G (2007). Forward and reverse hippocampal place-cell sequences during ripples. Nat Neurosci.

[21] El-Gaby M, Reeve HM, Lopes-Dos-Santos V, Campo-Urriza N, Perestenko PV, Morley A, Strickland LAM, Lukács IP, Paulsen O, Dupret D (2021). An emergent neural coactivity code for dynamic memory. Nat Neurosci.

[22] Fauth MJ, van Rossum MC (2019). Self-organized reactivation maintains and reinforces memories despite synaptic turnover. eLife.

[23] Fernández-Ruiz A, Oliva A, Soula M, Rocha-Almeida F, Nagy GA, Martin-Vazquez G, Buzsáki G (2021) Gamma rhythm communication between entorhinal cortex and dentate gyrus neuronal assemblies. Science 372.10.1126/science.abf3119 10.1126/science.abf3119 PMC8285088 33795429

[24] Folschweiller S, Sauer J-F (2022). Phase-specific pooling of sparse assembly activity by respiration-related brain oscillations. J Physiol.

[25] Foster DJ, Wilson MA (2006). Reverse replay of behavioural sequences in hippocampal place cells during the awake state. Nature.

[26] Gerstein GL, Bedenbaugh P, Aertsen AMHJ (1989). Neuronal assemblies. IEEE Trans Biomed Eng.

[27] Girin B, Juventin M, Garcia S, Lefèvre L, Amat C, Fourcaud-Trocmé N, Buonviso N (2021). The deep and slow breathing characterizing rest favors brain respiratory-drive. Sci Rep.

[28] González J, Torterolo P, Tort AB (2022) Mechanisms and functions of respiration-driven gamma oscillations in the piriform cortex. biorXiv. 10.1101/2022.04.24.489324 10.7554/eLife.83044 PMC10069865 36806332

[29] Grosmaitre X, Santarelli LC, Tan J, Luo M, Ma M (2007). Dual functions of mammalian olfactory sensory neurons as odor detectors and mechanical sensors. Nat Neurosci.

[30] Hammer M, Schwale C, Brankačk J, Draguhn A, Tort ABL (2021) Theta-gamma coupling during REM sleep depends on breathing rate. Sleep:zsab189. 10.1093/sleep/zsab189 10.1093/sleep/zsab189 34297128

[31] Harris KD (2005). Neural signatures of cell assembly organization. Nat Rev Neurosci.

[32] Hebb DO (1949). The organization of behavior.

[33] Heck DH, Kozma R, Kay LM (2019). The rhythm of memory: how breathing shapes memory function. J Neurophysiol.

[34] Henze DA, Buzsáki G (2001). Action potential threshold of hippocampal pyramidal cells in vivo is increased by recent spiking activity. Neuroscience.

[35] Herrero JL, Khuvis S, Yeagle E, Cerf M, Mehta AD (2018). Breathing above the brain stem: volitional control and attentional modulation in humans. J Neurophysiol.

[36] Ito J, Roy S, Liu Y, Cao Y, Fletcher M, Lu L, Boughter JD, Grün S, Heck DH (2014). Whisker barrel cortex delta oscillations and gamma power in the awake mouse are linked to respiration. Nat Commun.

[37] Jung F, Witte V, Yanovsky Y, Klumpp M, Brankačk J, Tort ABL, Draguhn A (2022). Differential modulation of parietal cortex activity by respiration and θ oscillations. J Neurophysiol.

[38] Karalis N, Dejean C, Chaudun F, Khoder S, Rozeske RR, Wurtz H, Bagur S, Benchenane K, Sirota A, Courtin J, Herry C (2016). 4-Hz oscillations synchronize prefrontal-amygdala circuits during fear behavior. Nat Neurosci.

[39] Karalis N, Sirota A (2022). Breathing coordinates cortico-hippocampal dynamics in mice during offline states. Nat Commun.

[40] Kluger DS, Balestrieri E, Busch NA, Gross J (2021). Respiration aligns perception with neural excitability. Elife.

[41] Kluger DS, Gross J (2021). Respiration modulates oscillatory neural network activity at rest. PLoS Biol.

[42] Koch C, Rapp M, Segev I (1996). A brief history of time (constants). Cereb Cortex.

[43] Kőszeghy Á, Lasztóczi B, Forro T, Klausberger T (2018). Spike-timing of orbitofrontal neurons is synchronized with breathing. Front Cell Neurosci.

[44] Larkum ME, Nevian T, Sandler M, Polsky A, Schiller J (2009). Synaptic integration in tuft dendrites of layer 5 pyramidal neurons: a new unifying principle. Science.

[45] Liu Y, McAfee SS, Heck DH (2017). Hippocampal sharp-wave ripples in awake mice are entrained by respiration. Sci Rep.

[46] Lockmann ALV, Laplagne DA, Leão RN, Tort ABL (2016). A respiration-coupled rhythm in the rat hippocampus independent of theta and slow oscillations. J Neurosci.

[47] Lopes-dos-Santos V, Conde-Ocazionez S, Nicolelis MAL, Ribeiro ST, Tort ABL (2011). Neuronal assembly detection and cell membership specification by principal component analysis. PLoS ONE.

[48] Lopes-dos-Santos V, Ribeiro S, Tort ABL (2013). Detecting cell assemblies in large neuronal populations. J Neurosci Methods.

[49] Lopes-Dos-Santos V, van de Ven GM, Morley A, Trouche S, Campo-Urriza N, Dupret D (2018). Parsing hippocampal theta oscillations by nested spectral components during spatial exploration and memory-guided behavior. Neuron.

[50] Markram H, Lübke J, Frotscher M, Sakmann B (1997). Regulation of synaptic efficacy by coincidence of postsynaptic APs and EPSPs. Science.

[51] McNaughton BL, Morris RGM (1987). Hippocampal synaptic enhancement and information storage within a distributed memory system. Trends Neurosci.

[52] Moberly AH, Schreck M, Bhattarai JP, Zweifel LS, Luo W, Ma M (2018). Olfactory inputs modulate respiration-related rhythmic activity in the prefrontal cortex and freezing behavior. Nat Commun.

[53] Nádasdy Z, Hirase H, Czurkó A, Csicsvari J, Buzsáki G (1999). Replay and time compression of recurring spike sequences in the hippocampus. J Neurosci.

[54] Nakamura NH, Fukunaga M, Oku Y (2018). Respiratory modulation of cognitive performance during the retrieval process. PLoS ONE.

[55] Nguyen Chi V, Müller C, Wolfenstetter T, Yanovsky Y, Draguhn A, Tort ABL, Brankačk J (2016). Hippocampal respiration-driven rhythm distinct from theta oscillations in awake mice. J Neurosci.

[56] Oberto VJ, Boucly CJ, Gao H, Todorova R, Zugaro MB, Wiener SI (2022). Distributed cell assemblies spanning prefrontal cortex and striatum. Curr Biol.

[57] Palm G, Knoblauch A, Hauser F, Schüz A (2014). Cell assemblies in the cerebral cortex. Biol Cybern.

[58] Paulk AC, Kfir Y, Khanna AR, Mustroph ML, Trautmann EM, Soper DJ, Stavisky SD, Welkenhuysen M, Dutta B, Shenoy KV, Hochberg LR, Richardson RM, Williams ZM, Cash SS (2022). Large-scale neural recordings with single neuron resolution using neuropixels probes in human cortex. Nat Neurosci.

[59] Pérez-Ortega J, Alejandre-García T, Yuste R (2021). Long-term stability of cortical ensembles. Elife.

[60] Peyrache A, Khamassi M, Benchenane K, Wiener SI, Battaglia FP (2009). Replay of rule-learning related neural patterns in the prefrontal cortex during sleep. Nat Neurosci.

[61] Sakurai Y, Nakazono T, Ishino S, Terada S, Yamaguchi K, Takahashi S (2013). Diverse synchrony of firing reflects diverse cell-assembly coding in the prefrontal cortex. J Physiol Paris.

[62] Schiller J, Major G, Koester HJ, Schiller Y (2000). NMDA spikes in basal dendrites of cortical pyramidal neurons. Nature.

[63] Schomburg EW, Fernández-Ruiz A, Mizuseki K, Berényi A, Anastassiou CA, Koch C, Buzsáki G (2014). Theta phase segregation of input-specific gamma patterns in entorhinal-hippocampal networks. Neuron.

[64] Shaw GL, Harth E, Scheibel AB (1982). Cooperativity in brain function: assemblies of approximately 30 neurons. Exp Neurol.

[65] Steinmetz NA, Zatka-Haas P, Carandini M, Harris KD (2019). Distributed coding of choice, action and engagement across the mouse brain. Nature.

[66] Stuart GJ, Spruston N (2015). Dendritic integration: 60 years of progress. Nat Neurosci.

[67] Tort ABL, Hammer M, Zhang J, Brankačk J, Draguhn A (2021). Temporal relations between cortical network oscillations and breathing frequency during REM sleep. J Neurosci.

[68] Tort ABL, Ponsel S, Jessberger J, Yanovsky Y, Brankačk J, Draguhn A (2018). Parallel detection of theta and respiration-coupled oscillations throughout the mouse brain. Sci Rep.

[69] van de Ven GM, Trouche S, McNamara CG, Allen K, Dupret D (2016). Hippocampal offline reactivation consolidates recently formed cell assembly patterns during sharp wave-ripples. Neuron.

[70] Yanovsky Y, Ciatipis M, Draguhn A, Tort ABL, Brankačk J (2014). Slow oscillations in the mouse hippocampus entrained by nasal respiration. J Neurosci.

[71] Zelano C, Jiang H, Zhou G, Arora N, Schuele S, Rosenow J, Gottfried JA (2016). Nasal respiration entrains human limbic oscillations and modulates cognitive function. J Neurosci.

[72] Zhang JX, Harper RM, Frysinger RC (1986). Respiratory modulation of neuronal discharge in the central nucleus of the amygdala during sleep and waking states. Exp Neurol.

[73] Zhong W, Ciatipis M, Wolfenstetter T, Jessberger J, Müller C, Ponsel S, Yanovsky Y, Brankačk J, Tort ABL, Draguhn A (2017). Selective entrainment of gamma subbands by different slow network oscillations. Proc Natl Acad Sci U S A.

